# Bioaccessibility-Based Fuzzy Health Risk Assessment and Integrated Management of Toxic Metals Through Multimedia Environmental Exposure near Urban Industrial Complexes

**DOI:** 10.3390/toxics13100861

**Published:** 2025-10-11

**Authors:** Siqi Xu, Donghua Zhu, Miao An, Haoyu Wang, Jinyuan Guo, Yazhu Wang, Yongchang Wei, Fei Li

**Affiliations:** 1 Research Center for Environment and Health, Zhongnan University of Economics and Law, Wuhan 430073, China; 2 School of Business Administration, Zhongnan University of Economics and Law, Wuhan 430073, China

**Keywords:** multimedia environment, urban toxic metals, bioaccessibility, fuzzy method, health risk assessment

## Abstract

Few studies have explored the holistic public health risk assessment associated with toxic elements (TEs) and their bioaccessibility in integrated urban environmental media including soils, vegetables, atmospheric particles, dust, etc. Urban industrial complex areas like Qingshan-Chemical District (QCD) in the Chinese Wuhan city, located within the Yangtze River Economic Belt, face increasing environmental exposure risks due to industrial activities. This study innovatively assessed the hierarchical risks of toxic metals in 4 environmental media (air PM, dust, soil, vegetables) from the QCD based on field sampling and chemical analysis, and developed an improved fuzzy health risk assessment model based on toxic metals’ in vitro bioaccessibilities of different exposure pathways and triangular fuzzy numbers for handling parameter uncertainties. The study found that the highest health risks were associated with ingestion, particularly from consuming homegrown vegetables. Carcinogenic risks for arsenic (As), lead (Pb), and cadmium (Cd) via ingestion exceeded the admissible threshold of 1.00 × 10^−6^, with As showing the highest risk ([1.92 × 10^−3^, 2.37 × 10^−3^]), followed by Cd ([2.98 × 10^−5^, 3.67 × 10^−5^]) and Pb ([7.92 × 10^−7^, 1.48 × 10^−6^]). Inhalation risks from soil, dust, and air particulates were below the threshold, indicating lower respiratory concerns. Dermal exposure, especially from soil and dust, posed elevated carcinogenic risks for As ([7.47 × 10^−6^, 8.06 × 10^−6^]). With the screened priority risk control toxic metals and pathways, the targeted measures including relocating vegetable planting areas, promoting cultivation of low-enrichment crops, building vegetation buffer zones around the industrial park, etc., were proposed.

## 1. Introduction

More than 50% of the worldwide population currently resides in urban zones. These regions have emerged as the primary geographic hubs for resource utilization and chemical discharges. Among these urban centers, industrial complexes function as a type of industrial symbiotic system and have been spread extensively globally [1]. Several factories may cluster in a given area, forming industrial complexes or specialized administrative zones, including farmland, residential land, etc. Generally, contaminants in the environmental multi-media (such as water, soil, air, and foods) of urban industrial complexes have higher contaminated background levels than those of other regions. Pollutants are intensifying, leading to chronic public health risks and an exacerbation of health hazards via multi-exposure routes and heterogeneous distribution. Among the various pollutants contained in environmental media, the presence of toxic metals (TMs) attracted crucial attention on account of the persistent, bioavailable, and toxic properties, which posed adverse influences on ecosystems and human well-being [2]. Globally, over 20 million hectares of land have been identified as polluted, with over 50% affected by toxic elements (TEs) [3,4]. As we all know, urban soils serve as a central functional component within regional environmental systems, acting as a critical hub for the exchange of materials and energy between the atmosphere, hydrosphere, biosphere, and lithosphere [5,6]. So, it is essential to explore the public health risk of TMs through multimedia environmental exposure near urban industrial complexes and draft a targeted management policy.

Over the past few years, scholars have extensively investigated the distribution and sources of TMs across various urban environmental media, including water [7,8], soil [9,10], and dust [11,12], etc. Some of those studies have also explored the bioaccessibility of TMs according to their chemical forms [13,14]. Additionally, some studies have focused on the correlation with soil physicochemical properties [15,16], ecological and health risk levels [17,18], primary sociometric factors contributing to contamination [19,20], and the development of corresponding remediation technologies [21,22]. Now, the process of exposure health risk assessment acts as a vital method for comprehensively characterizing and managing the detrimental influences of the TEs across environmental (multi)media. Unfortunately, existing environmental quality and risk management for TEs in soils, vegetables, and atmospheric particulates commonly re-main relatively independent and fail to consider the mobility of TEs across different environmental media, which poses a significant challenge for a holistic risk management especially in areas with mixed land use areas [23]. Some studies have explored improved exposure risk assessment models that account for the bioaccessibility of TEs in various environmental media, such as soils, dust, and atmospheric particulates, highlighting the risk of over- or under-estimation in health risk judgments. Given the primary exposure routes, including oral ingestion, inhalation, and dermal contact, it is critical to employ appropriate scientific methods for the quantitative analysis of toxic metal (TM) bioaccessibility across these pathways. Currently, there are no rules and regulations to establish a global method standard for TM bioaccessibility. In comparison with some sequential extraction procedures (Tessier method [24] and BCR method [25]) which could only indicate biological migration from soil/atmospheric particulates to plants, some in vitro methods, including Simulating Lung Fluid Experiment [26,27], Simple Bioaccessibility Extraction Test (SBET) [28,29], Physiologically Based Extraction Test (PBET) [30], in Vitro Skin Permeation Test [31], Simulated Intestinal Fluid [32], etc., were used to make quantitative evaluation of pollutants’ bioaccessibility in different target exposure scenarios. Obviously, differing theoretical bases can lead to variations in evaluation outcomes and conclusions, potentially perplexing policymakers or misleading policy outcomes. Hence, it is significant to establish a scientific and feasible health risk-based integrated assessment and management framework with an integrating consideration with TMs’ toxicity and bioaccessibility on different exposure pathways in environmental multimedia, together with good parameter uncertainty control.

The Qingshan-Chemical District (QCD) in Wuhan, China, serves as a notable example of Industrial Symbiosis, featuring a historical steel industrial complex and an ethylene chemical industrial complex [4]. In recent years, some related research efforts have been conducted in QCD, including monitoring TEs in the soil of QCD [33,34], evaluating health risk on dust TMs [12], or on the vegetable bioaccumulation [35], while few studies have carried out a holistic public health risks posed by TEs among exposure environmental multimedia. Hence, the major aims of this study were to (i) investigate TEs concentrations in major exposure environmental multimedia including soils, vegetables, atmospheric particles, dust, drinking waters; (ii) measure TEs bioaccessibility levels using methods related to exposure features of corresponding pathway, for example, Simulating lung fluid (SLF) method for atmospheric particles; (iii) establish a novel integrated fuzzy health risk assessment integrating TMs’ toxicity and bioaccessibility; and (iv) perform a local multimedia health risk assessment and propose an integrated management policy.

## 2. Materials and Methods

### 2.1. Study Area and Multimedia Environment Sampling

The Qingshan Chemical District (QCD), located in the eastern part of the city of Wuhan, serves as a key heavy industrial hub in the mid-Yangtze River economic belt. The study area is known as the North Lake region, with geographic coordinates between 30.62–30.68° N latitude and 114.47–114.54° E longitude. The area is situated between two major industrial complexes, China Baowu Iron and Steel Group Co., Ltd. (Wuhan, China) and China-Korea (Wuhan) Petrochemical Co., Ltd. (Wuhan, China), which constitute the 2 pillar industries of the QCD. QCD was selected as a typical area in the Yangtze River Economic Zone to conduct a practical study to actively explore the all-element risk identification mechanism of TM exposure in its industrial complex area, and to scientifically explore the risk wisdom dynamic management of cross-media TM exposure, to provide the realization details and practical experience for the validation of the region’s all-element environmental health risk wisdom management and control decision-making system.

This plan involves multi-temporal and spatial-scale sampling and analysis of atmospheric particulate matter, with a particular focus on various occupational exposure groups and traffic conditions. The precise coordinates of sampling points are detailed in Table 1. Particulate matter samples of varying particle sizes were collected across the entire Qingshan District during 4 seasons (spring, summer, autumn, and winter). Under clear weather conditions, 4 outdoor particulate matter samplers were deployed for continuous 12 h sampling. Instrument specifications are outlined in Table S1. Post-collection, filter membranes were returned to the laboratory for drying to constant weight, weighing, and division processing before being sealed for storage. A total of 75 valid atmospheric particulate matter samples were obtained. Specific outdoor sampling locations included roadside areas, traffic police booths, school entrances, and public squares.

**Figure 1:**
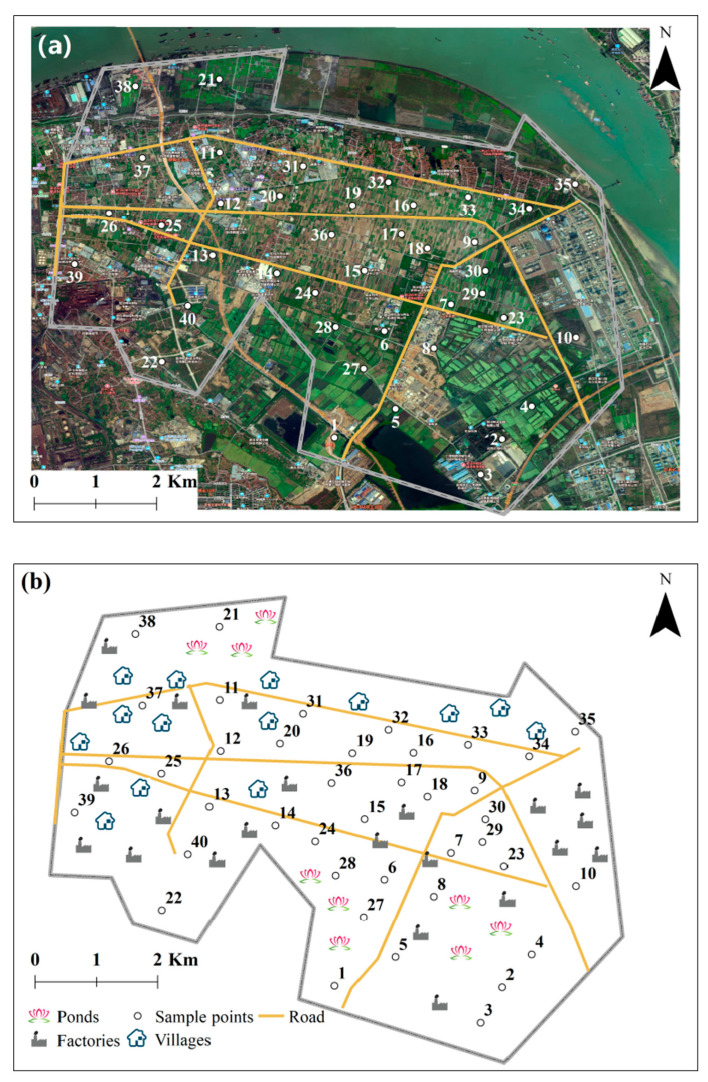
Satellite imagery (**a**) and schematic diagram (**b**) of sample points.

A total of 59 surface soil samples were gathered from designated locations and subsequently blended to form a representative composite sample. In order to ensure comprehensive coverage of the agricultural planting areas within the study region, the spatial arrangement of these sampling points, which followed a 1 km × 1 km grid layout with adjustments for ponds and factories, is illustrated in Figure 1.

Farmers surveyed indicated that the North Lake area was a vegetable supplier to Wuhan and other nearby cities. In this study, common vegetables’ edible parts were collected from agricultural fields for analysis, including leafy vegetables (bok choy and amaranth greens), stem vegetables (water spinach and tender flower stalk), and root vegetables (radish). The vegetable samples collected should be at the mature stage. When collecting vegetable samples, at least 3 plants of the same kind were selected in a 50 m × 50 m area to mix to make one mixed sample, and were recorded at the same time.

The dust sample collection in the Qing Shan District of Wuhan City was conducted from the winter to the autumn, covering spring, summer, autumn, and winter. Dust samples were collected on clear, windless days from various impervious surfaces near 27 sampling points (1, 5, 6, 7, 8, 9, 10, 11, 12, 13, 14, 16, 17, 19, 22, 23, 24, 25, 26, 31, 32, 33, 34, 37, 38, 39, 40), which were selected from among the 40 sampling sites. The samples were then sifted to remove impurities before storage.

### 2.2. Pre-Treatment and Sample Analysis

#### 2.2.1. Atmospheric Particles and Dust

Atmospheric particulate matter collected was dried and weighed to remove moisture to ensure accurate quality of the particulate matter for subsequent analysis of TM concentrations and chemical composition. Dust samples collected from the ground were sieved to remove impurities and stored properly. Atmospheric particulate matter was exposed to simulated lung fluid to simulate the human lung fluid environment, while dust was exposed to simulated lung fluid and simulated gastric fluid to simulate the human lung environment and stomach digestion environment. The soluble TMs contained therein were extracted by the above methods.

Simulating lung fluid (SLF), referring to the proportions by Pavel Coufalík [36,37], the solution contains sodium chloride (3.210 g·L^−1^), sodium hydroxide (6.000 g·L^−1^), citric acid (20.8000 g·L^−1^), calcium chloride dihydrate (0.1285 g·L^−1^), sodium citrate dihydrate (0.077 g·L^−1^), sodium phosphate dodecahydrate (0.2347 g·L^−1^), sodium sulfate (0.039 g·L^−1^), magnesium chloride hexahydrate (0.106 g·L^−1^), glycerol (0.059 g·L^−1^), sodium tartrate dihydrate (0.090 g·L^−1^), sodium lactate (0.085 g·L^−1^), sodium pyruvate (0.086 g·L^−1^) and formaldehyde (1 mL·L^−1^). Take 2 portions of 1/4 membrane samples, cut them into small pieces, and place them in two 50 mL centrifuge tubes. Add 20 mL SLF to each, and oscillate at 200 rpm for 24 h. Filter, and take 10 mL of supernatant into a 10 mL centrifuge tube.

Simulating gastric fluid, using simulated SBET for the stomach, first prepare a 0.4 M glycine solution and adjust the pH to 1–2 with hydrochloric acid. Take about 0.5 g of dust sample, place it in a centrifuge tube containing 50 mL of the solution, and shake it in a thermostatic box at 37 °C and 100 rpm for 1 h. Add a small amount of ferrous sulfate solution to maintain anaerobic conditions. After completing the above process, filter the solution with a 0.45 μm membrane filters, add an appropriate amount of hydrochloric acid to ensure acidification, and store it in a refrigerator for testing.

TMs and anions in samples were measured according to national standards using various methods such as atomic absorption spectrophotometry and ion chromatography.

#### 2.2.2. Soil and Vegetables

Soil samples were dried, crushed, and screened through sieves of different mesh sizes. The samples were then divided, with one portion reserved and another ground finely for analysis. Vegetables were cleaned, dried, and then ground into a powder for testing. TMs in soil and vegetables were detected using advanced instruments after sample digestion with acids. Various extraction methods were used to analyze different fractions of metals in the soil.

The TMs in soil, dust, and vegetables were determined using the ThermoICAP-Q system from the United States. The sample digestion process was as follows: accurately weigh 0.1 g of solid sample and place it in a cleaned polytetrafluoroethylene (PTFE) digestion vessel. Add 15 mL of nitric acid and 2 mL of hydrochloric acid, seal the vessel, and run the microwave digestion program. After digestion is complete, transfer the sample to a clean polyester (PET) plastic bottle and dilute to a mass of 50.00 g with ultrapure water. The blank control was treated in the same manner. The PBET method was used to simulate human digestion and assess the bioaccessibility of TMs in vegetables. This process included stages simulating gastric and intestinal conditions, with measurements taken after each stage.

All laboratory equipment, including plastic materials and glassware, underwent a thorough cleaning procedure. They were first soaked in a 20% nitric acid solution overnight to ensure any contaminants were effectively removed. Following this, they were rinsed with deionized water prepared by Barnstead GenPure water purification system (Thermo Fisher Scientific, Waltham, MA, USA) to prepare them for use in experiments. To maintain high standards of quality control during the analysis, we incorporated control samples, standard reference materials, and parallel samples [38] into our testing protocol at a ratio of 10%. This practice ensured that our results adhered strictly to established test criteria. Specifically, the reliability of trace element analyses was confirmed when the error between repeated sample analyses remained under 5%, demonstrating acceptable analytical precision within a ±10% range for replicate samples. Moreover, the recovery rates for the reference samples fell within an optimal range of 90% to 110%, indicating the accuracy and consistency of our methods.

### 2.3. Bioaccessibility-Based Fuzzy Health Risk Assessment Method

**Figure 2:**
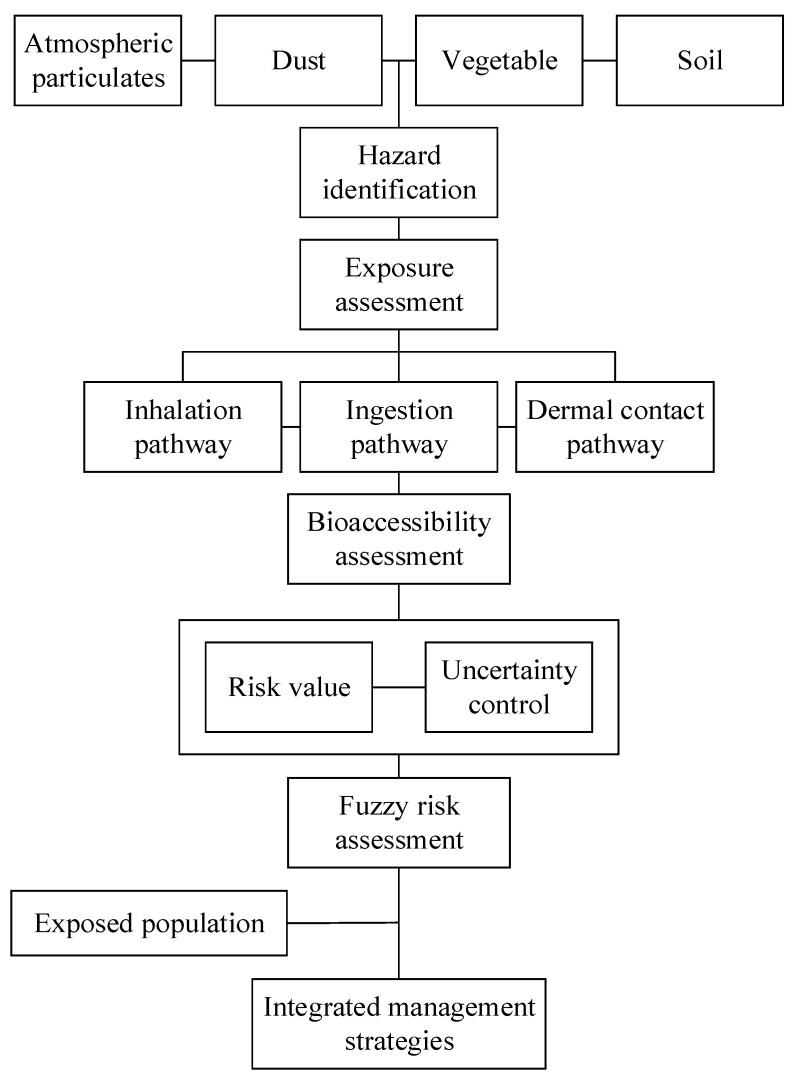
Bioaccessibility-based fuzzy health risk assessment workflow.

According to the Technical Guidelines for Ecological and Environmental Health Risk Assessment in China [39], the core stages of the assessment methodology are clearly defined. This comprises several key stages: hazard identification and characterization (Table S2), exposure analysis, risk evaluation, and uncertainty management [4]. The methodological framework that guided the fuzzy health risk assessment was depicted in Figure 2. In the following sections, we would delve deeper into each step of the assessment process, providing a thorough examination of the methodologies and approaches used.

#### 2.3.1. Health Risk Characterization

The bioaccessibility proportion (BA%) of each metal during the gastrointestinal stage is calculated using the following formula:

(1) $$BA=\frac{C_{extraction\;concentration}}{C_{total\;concentration}}\times 100\%$$

(1) Oral ingestion

For the residents of Qing Shan District, the main exposure pathways of TM oral ingestion are divided into dust and vegetables.

(2) $${ADD}_{D(ing)}=C\times {BA}_{D}\times \frac{{Ing}_{R}\times EF\times ED}{BW\times AT}\times 10^{-6}$$

(3) $${ADD}_{V(ing)}=C\times {BA}_{V}\times \frac{IR\times ED\times EF\times RSP}{BW\times AT}\times 10^{-3}$$

where ADD_D(ing)_ and ADD_V(ing)_ are the daily mean exposure doses of the receptors via ingestion pathways of dust and local vegetables, respectively, with units of mg·g^−1^·d^−1^, detailed information on all relevant parameters can be found in Table S3.

(2) Inhalation

For the residents of Qing Shan District, the main pathways of TM inhalation exposure are divided into atmospheric particulate matter and dust.

(4) $${ADD}_{PM(inh)}=C\times {BA}_{PM}\times \frac{ED\times EF\times InhR}{BW\times AT}$$

(5) $${ADD}_{D(inh)}=C\times {BA}_{D}\times \frac{InhR\times EF\times ED}{PEF\times BW\times AT}$$

where ADD_PM(inh)_ and ADD_D(inh)_ are the daily average exposure doses of the receptors through inhalation pathways of atmospheric respirable particulate matter and dust, respectively, with units of mg·kg^−1^·d^−1^, detailed information on all relevant parameters can be found in Table S3.

(3) Dermal contact

For the residents of Qing Shan District, the main pathway of TM exposure through dermal contact is primarily through dust.

(6) $${ADD}_{D(der)}=C\times {BA}_{D}\times \frac{SA\times AF\times ABS\times EF\times ED}{BW\times AT}\times 10^{-6}$$

where ADD_D(der)_ represents the daily mean dose of the receptor through dermal contact with dust, with units of mg·kg^−1^·d^−1^, detailed information on all relevant parameters can be found in Table S3.

For soils, the biotoxicity of TMs is influenced not only by their total concentration but also, to a greater extent, by their chemical fractions. To assess the bioaccessibility of different chemical fractions of TMs in soils, a hierarchical extraction method was employed, which ranks the bioaccessibility of these fractions as follows: exchangeable fraction > carbonate-bound fraction > iron-manganese oxide-bound fraction > organic complexed fraction > residual fraction. Therefore, the hierarchical relationship between the components of TMs and bioaccessibility is adjusted as follows:

(7) $$C_{bio}=C_{i}\times (f_{S1}+f_{S2})$$

(8) $$C_{pbio}=C_{i}\times (f_{S3}+f_{S4})$$

(9) $$C_{Nbio}=C_{i}\times f_{S5}$$

where $f_{S1}$, $f_{S2}$, $f_{S3}$, $f_{S4}$, and $f_{S5}$ are the percentages of the total metal concentration that are associated with the exchangeable fraction (S1), carbonate-bound fraction (S2), iron-manganese oxide-bound fraction (S3), organic complexed fraction (S4), and residual fraction (S5), respectively. $C_{\mathrm{bio}},\;C_{\mathrm{pbio}}$, and $C_{\mathrm{Nbio}}$ are the concentrations of the bioavailable, potentially bioavailable, and non-bioavailable components in the corresponding samples, respectively. Finally, the TM concentrations are triangularly fuzzy as ($C_{\mathrm{bio}},\;C_{\mathrm{pbio}}$)_min_, $C_{i}$, ($C_{\mathrm{bio}},\;C_{\mathrm{pbio}}$)_max_.

The calculation formulas for the exposure of 4 TMs (Cd, As (inorganic), Pb, and Hg) in soil are as follows:

(10) $${ADD}_{S(ing)}=\frac{C_{soil}\times OSIR\times ED\times EF\times {ABS}_{O}}{BW\times AT}\times 10^{-6}$$

(11) $${ADD}_{S(inh)}=\frac{C_{s}\times PM\times DAIR\times ED\times PIAF\times EF\times (f_{spo}\times EFO+f_{spi}\times EFI)}{BW\times AT}\times 10^{-6}$$

(12) $${ADD}_{S(der)}=\frac{C\times TSA\times SER\times SSAR\times EF\times ED\times E_{V}\times {ABS}_{d}}{BW\times AT}\times 10^{-6}$$

where ADD_S(ing)_, ADD_S(inh)_, ADD_(Sder)_ are the daily mean doses of the receptor through 3 previously mentioned pathways from soil, respectively, with units of mg·kg^−1^·d^−1^, detailed information on all relevant parameters can be found in Table S3.

The non-carcinogenic and carcinogenic risks associated with metal element i through exposure route j were assessed using the following equations:

(13) $$HQ=\frac{{ADD}_{ing/inh/dermal}}{RfD}$$

(14) $$\mathrm{CR}={\mathrm{ADD}}_{\mathrm{ing}/\mathrm{inh}/\mathrm{dermal}}\times \mathrm{SF}$$

where ADD_ing_, ADD_inh_, ADD_dermal_ are the daily average doses of the receptor via inhalation of atmospheric particulates, oral ingestion and dermal contact with dust, vegetables, and soil, respectively, in units of mg·kg^−1^·d^−1^; The Hazard Quotient (HQ) is a unitless metric used to assess non-carcinogenic risk. Similarly, the Carcinogenic Risk (CR) is also expressed as a unitless value. The Reference Dose (*RfD*) for non-carcinogenic risk is given in mg·kg^−1^·d^−1^. Slope Factor (SF) for carcinogenic risk is expressed in terms of kg·d·mg^−1^.

#### 2.3.2. Uncertainty Control Based on TFN

Given the intricate and uncertain nature of assessing environmental health risks, using the mean concentration and single values of parameters from experimental analysis to calculate and evaluate using the above formulas might lead to biased conclusions [40]. Both triangular fuzzy numbers (TFNs) and Monte Carlo simulation are commonly used methods for controlling uncertainty. While Monte Carlo simulation is highly effective in handling variability and randomness with sufficient data, it often requires large sample sizes and precise probability distributions to accurately reflect real-world variability [41,42]. In contrast, TFNs are particularly advantageous in dealing with epistemic uncertainty arising from sparse data, vague parameters, or limited sampling resources [43]. Given the cost constraints and limited sample size in this study, TFNs provides a more feasible and robust approach to capture the inherent vagueness and imprecision in exposure parameters, without the need for extensive data.

The definition of a triangular fuzzy number was as follows: Let Ã^α^ (a, b, c) be a fuzzy number defined in the real number domain R. The membership function μÃ (x) of the function had a value range of [0, 1] [44,45]:

(15) $$\mu Ã\left(x\right)=\left\{\begin{array}{c} 0,\;\;\;\;\;\;\;\;\;\;\;\;\;x<a\;or\;x>c\\ \frac{x-a}{b-a},\;\;\;\;\;\;\;\;\;a\leq x\leq b\;\;\;\;\\ \frac{c-x}{c-b},\;\;\;\;\;\;\;\;\;b\leq x\leq c\;\;\;\; \end{array}\right.$$

Among them, a, b, and c are non-negative real numbers, respectively, representing the minimal value, the expected value, and the maximal value. These values are used to construct the triangular fuzzy number (a, b, c). To simplify the calculation, the α-cut technique is employed. The calculation process is as follows:

(16) $${Ã}^{\alpha }\;=\;[a_{L}^{\alpha },a_{R}^{\alpha }]\;=\;[\left(b-a\right)\alpha +a,\;-(c-b)\alpha +c]$$

where α denotes the confidence level, with α = 0.9 being an acceptable threshold for reliability. Consequently, all α-cutoff analyses in this study employ α = 0.9 [46,47]. The fuzzy interval of the fuzzy number Ã at the specified confidence level is denoted by ${Ã}^{\alpha }$. The rules for the arithmetic operations of fuzzy numbers are as follows [48]:

(17) $${Ã}_{1}^{\alpha }+{Ã}_{2}^{\alpha }=\;[a_{L1}^{\alpha }+a_{L2}^{\alpha },\;a_{R1}^{\alpha }+a_{R2}^{\alpha }]$$

(18) $${Ã}_{1}^{\alpha }÷{Ã}_{2}^{\alpha }=[a_{L1}^{\alpha }÷a_{L2}^{\alpha },\;a_{R1}^{\alpha }÷a_{R2}^{\alpha }]$$

(19) $${Ã}_{1}^{\alpha }\times {Ã}_{2}^{\alpha }=[a_{L1}^{\alpha }\times a_{L2}^{\alpha },\;a_{R1}^{\alpha }\times a_{R2}^{\alpha }]$$

(20) $${kÃ}_{1}^{\alpha }=[{ka}_{L1}^{\alpha },\;ka_{R1}^{\alpha }]$$

Based on Equations (1)–(20), final fuzzy intervals for health risks were obtained. If HQ > 1, it is considered that there is a non-carcinogenic health risk. If HQ < 1, the non-carcinogenic risk is considered small or negligible. The standard for evaluating the carcinogenic risk of chemical substances is the “maximum acceptable level”, which is set at 5 × 10^−4^ by the International Commission on Radiological Protection (ICRP) and 1 × 10^−6^ in China, while the Swedish Environmental Protection Agency gives the maximum acceptable and negligible risk value of 1 × 10^−6^ (7 × 10^−5^), and the U.S. Environmental Protection Agency (USEPA) considers all risk values within the 1 × 10^−4^ level to be acceptable, and USEPA considers all risk values within the 1 × 10^−4^ level to be acceptable. There is some variability in the limit values of different evaluation criteria. With the help of the expert consultation method, this report classified the carcinogenic risk level into 7 classes regarding the Guidelines [49], the evaluation guidelines of the USEPA and ICRP, and relevant literature, and the evaluation criteria were presented in Table S4.

## 3. Results and Discussion

### 3.1. TMs in QCD Multimedia Environment

#### 3.1.1. Atmospheric Particulates

The collected PM2.5, PM5, PM10, and TSP samples were subjected to analysis for arsenic (As), cadmium (Cd), lead (Pb), and nickel (Ni), representing a total of 4 TMs. The content of TMs in the atmospheric particulates in the air particles of the QCD from the winter to the autumn (C_PMM_), and the concentration of TMs (C_M_) are detailed in Tables S5 and S6, respectively. Compared to the ambient air quality standards (NAAQS, [50]), the quarterly average secondary concentration limit for Pb is 1 × 10^−3^ mg·m^−3^, while the annual average limit is 5 × 10^−4^ mg·m^−3^. The annual mean secondary concentration limit for Cd is 5 × 10^−6^ mg·m^−3^, and for As, the annual average secondary concentration limit is 2.5 × 10^−8^ mg·m^−3^. From winter to autumn, Pb concentrations in PM2.5, PM5, PM10, and total suspended particles (TSP) remained consistently below national standards. However, Cd concentrations exceeded the standards at various rates: 12% of the time in PM2.5 (19.67 times the limit), 42% in PM5 (20.10 times), 50% in PM10 (20.37 times), and 82% in TSP (20.77 times). As concentrations consistently exceeded national standards across all particle sizes (PM2.5, PM5, PM10, and TSP), with a 100% exceedance rate. The exceedance multiples ranged from 1.34 to 5.69 times the limit for PM2.5, 1.52 to 5.43 times for PM5, 1.71 to 5.69 times for PM10, and 1.89 to 5.23 times for TSP. When compared to the World Health Organization (WHO) standards (6.6 ng m^−3^ for As), As levels also showed a 100% exceedance rate.

#### 3.1.2. Dust

Our research gathered dust samples from 8 locations in the QCD. Laboratory processing and chemical analysis revealed the TM content of these samples. Table 2 presents the descriptive statistics for the total concentrations of these metals (As, Cd, Pb, and Ni) in the dust samples.

As shown in Table 2, the mean contents of As, Cd, Pb, and Ni in dust were 3.88, 0.53, 78.41, and 35.20 mg·kg^−1^, respectively, and the mean concentrations in descending order were: Pb > Ni > As > Cd. Since there was no standard for the limits of TMs in dust, this research referred to the soil background values from Hubei Province, as documented in China’s Background Values of Soil Elements, and the risk screening thresholds outlined in the Soil Environmental Quality Standards for comparison. The TMs in dust, except As, exceeded the soil background values from Hubei Province. Cd, Pb, and Ni were 3.11, 2.94, and 1.21 times the soil background values, respectively. Pb exceeded the background value in all of the 4 sample points, while Cd exceeded these levels in 62.5% of cases. It suggested that Cd and Pb had more obvious anthropogenic enrichment situations. The coefficient of variation (C.V), which quantified the average variability of dust samples within the study area, was ordered as Ni (107%) >Pb (101%) > Cd (58%) > As (42%), which indicated that Ni and Pb were more affected by human activities and had obvious spatial distribution differences.

#### 3.1.3. Soil

In this study, 20 soil samples were collected from the QCD area, and the results of TM content in agricultural soils were obtained after laboratory treatment and chemical analysis. The descriptive statistics of the total concentration of TMs (As, Cd, Pb, Hg) in agricultural soils in the agricultural soils are shown in Table 3.

As illustrated in Table 3, the mean content of As, Cd, Pb, and Hg in the soil samples was 3.84 mg·kg^−1^, 0.24 mg·kg^−1^, 18.82 mg·kg^−1^, and 0.29 mg·kg^−1^, respectively. Comparing with the soil background values of soil TMs in Hubei Province, the ratios of these elements followed the order: Hg > Cd > Pb > As, in which all the soil samples exceeded the background value for Hg in Hubei Province, 60% and 10% of soil samples exceeded the background values for Cd and Pb in Hubei Province, respectively, while the As content of the soil within the study site did not exceed the background value of Hubei Province, which showed that Hg and Cd had a more obvious anthropogenic enrichment situation. By the Soil Environmental Quality Standard [51], the content of soil metal elements, including As, Cd, Pb, and Hg, at all sites remained below the corresponding risk screening values. The coefficient of variation quantified the variability of soil samples in the study area. A higher coefficient of variation indicated greater heterogeneity in TM distribution, which suggested a stronger influence from human production activities. According to Wilding’s study [52], when the coefficient of variation (C.V) was ≥36%, it was a high level of variability, when 16% ≤ C.V < 36%, it was an intermediate level of variability, and when C.V < 16%, it was a low level of variability. The coefficients of variation of the 4 metal elements were Pb (72%) > Cd (55%) > Hg (38%) > As (23%), and the C.V of Pb, Cd, and Hg belonged to the degree of high-level variation, which indicated that these 3 TMs were greatly attributable to human-related activities, and the differences in spatial distribution were obvious.

#### 3.1.4. Locally Grown Vegetables

The TM content of locally grown vegetables, including amaranth (AM), water spinach (WS), radish (RA), tender flower stalk (TFS), and bok choy (BC) in the North Lake area, is shown in Table S7. Comparison with the standards in the National Food Safety Standard Limits of Contaminants in Foods [53] indicated that the average TM concentrations in these 5 vegetables were within the permissible limits set by the national food safety standards. The order of As content (mg·kg^−1^) in the 5 kinds of vegetables was: WS (0.140, 0.08~0.190) > AM (0.030, 0.021~0.048) > TFS (0.023, 0.016~0.030) > BC (0.017, 0.010~0.024) > RA (0.007,0.006~0.007), indicating that As was more likely to accumulate in water spinach (WS). The order of Cd content (mg·kg^−1^) in the 5 kinds of vegetables was: AM (0.145, 0.062~0.357) > TFS (0.038, 0.011~0.083) > WS (0.033, 0.022~0.046) ≈ BC (0.033, 0.020~0.047) > RA (0.007, 0.006~0.007). Among them, the Cd content in amaranth at some sampling points was close to or even exceeded the limit, indicating that Cd in amaranth (AM) has potential risks. The order of Pb content (mg·kg^−1^) in the 5 kinds of vegetables was: flowering TFS (0.052, 0.007~0.088) > AM (0.048, 0.019~0.083) > BC (0.041, 0.027~0.047) > WS (0.029, 0.01~0.041) > RA (0.004, 0.002~0.009), indicating that Pb was more likely to accumulate TFS. The order of Hg content (mg·kg^−1^) in the 5 kinds of vegetables was: TFS (0.005, 0.001~0.007) > WS (0.003, 0.002~0.003) ≈ BC (0.003, 0.002~0.003) > AM (0.002, 0.001~0.003) > RA (0.001, 0.000~0.002). Moreover, the contents of the 4 TMs in radish were all relatively low, indicating that compared with other vegetables, radish was less likely to accumulate the 4 TMs of As, Cd, Pb, and Hg.

### 3.2. Bioaccessibility of TMs in a Multimedia Environment

In the following bioaccessibility comparisons across different media and metals, the mean values of bioaccessibility percentages are primarily used for statistical analysis and comparative assessment. The ranges or intervals are provided to indicate data variability.

#### 3.2.1. Atmospheric Particulates

**Figure 3:**
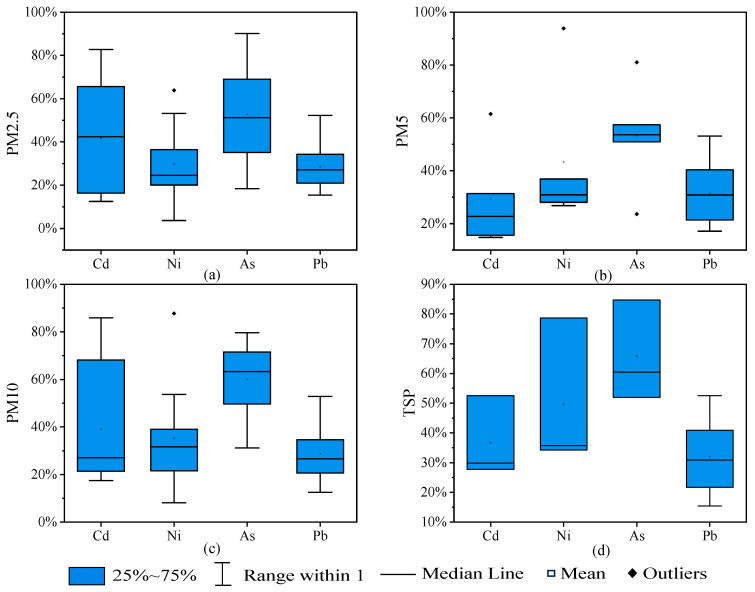
Bioaccessibility (BA_PM_) of heavy metal in PM2.5 (**a**), PM5 (**b**), PM10 (**c**), TSP (**d**).

The levels of biologically accessible TMs (C_PMBA_) in atmospheric particulate matter (PM) in the Green Mountain District for the period 2020 to 2021 are detailed in Table S8, and the content of biologically accessible TMs (C_BA_) in PM in the Green Mountain District for the period 2020 to 2021 are illustrated in Table S9. The biologically accessible properties of the metal elements (BA_PM_) in PM are detailed in Figure 3 and Table S10.

The bioaccessibility of Cd was highest in PM2.5 and followed a sequential increase in PM5, PM10, and TSP. For Ni, bioaccessibility also increased sequentially across PM2.5, PM5, PM10, and TSP. As had the lowest bioaccessibility in PM2.5 and the highest in PM10. Pb had the lowest bioaccessibility in PM10. Zn and Cu had the lowest bioaccessibility in PM2.5 and the highest in TSP. Overall, bioaccessibility was generally higher in PM2.5 and PM10 compared to PM5 and TSP for most TMs.

#### 3.2.2. Dust

**Figure 4:**
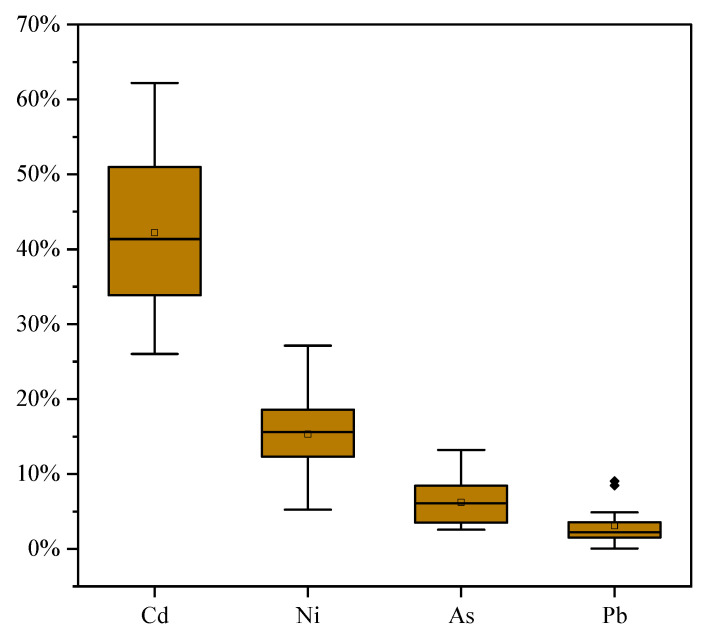
Bioaccessibility of TMs in dust (BA_D_).

The bioaccessibility (B_AD_) of TMs found in dust samples is presented in Figure 4, with specific data in Table S11. From winter 2020 through autumn 2021, the ranking of TM bioaccessibility in dust, from highest to lowest, was Cd (42.2%, 26.0~62.2%) > Ni (15.3%, 5.2~27.1%) > As (6.2%, 2.6~13.2%) > Pb (3.1%, 0.04~9.0%).

#### 3.2.3. Soil

The accumulation patterns of TMs are closely associated with their mobility, bioaccessibility, and biotoxicity, which are essential for assessing the degree of TM pollution and potential ecological risks. It is commonly acknowledged that TMs in the exchangeable and carbonate-bound forms are relatively unstable and more easily transported. In this study, an enhanced Tessier sequential extraction procedure was applied to identify the chemical fractions of TMs in soil samples.

**Figure 5:**
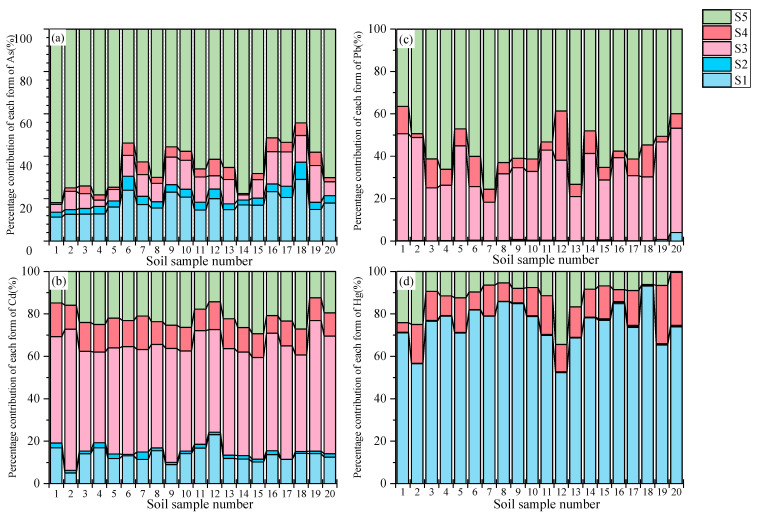
The contribution of the 5 fractions of As (**a**), Pb (**b**), Cd (**c**), Hg (**d**) to the total amount of TMs in soil (%).

Following statistical analysis, the contribution of the 5 fractions of the 4 TMs to the total amount of TMs is illustrated in Figure 5. For As, it was mainly in the residue state, and the overall distribution trend of chemical fractions of As in all sampling sites was as follows: S5 (64.94%, 44.27~81.85%) > S1 (17.76%, 11.30~29.10%) > S3 (9.59%, 2.40~17.31%) > S4 (3.97%, 0.69~7.83%) > S2 (3.75%, 2.20~8.11%). Cd in the soil was mainly dominated by S3, and the overall distribution trend of the chemical fraction of Cd was as follows: S3 (51.23%, 42.64~66.49%) > S5 (21.77%, 12.44~29.41%) > S1 (13.37%, 5.03~23.10%) > S4 (12.12%, 8.27~15.90%) > S2 (1.51%, 0~3.53%). Pb in the soil was also mainly dominated by S5, and the overall distribution trend of the chemical fraction of Pb was as follows: S5 (56.17%, 36.43~75.56%) > S3 (35.11%, 18.19~50.45%) > S4 (8.23%, 1.75~23.17%) > S1 (0.39%, 0~4.02%) > S2 (0.09%, 0~0.33%). Hg in the soil was mainly dominated by S1, and the overall distribution trend of the chemical fraction of Hg was as follows: S1 (75.16%, 52.40~93.60%) > S4 (13.15%, 0~27.42%) > S5 (11.43%, 0.48~34.47%) > S3 (0.23%, 0.05~0.51%) > S2 (0.20%, 0.05~0.56%).

Among them, Pb and As were predominantly in the residue state, which had limited mobility and was not readily released or taken up by plants, thereby posing minimal ecological risk to the surrounding environment. The average contribution rate of the exchangeable fraction of Hg in the soil reached 75.16%. This fraction exhibits high mobility, is highly sensitive to changes in environmental conditions, and can be readily absorbed by plants directly, indicating that mercury poses a significant potential ecological risk. While Cd in soil was mainly dominated by ferromanganese oxidation state (S3) (51.23%), this fraction will be indirectly absorbed by plants as pH decreases and TM ions are released under reducing conditions, resulting in a potential ecological risk. Additionally, it is readily absorbed by plants, which increases the potential ecological risk associated with Hg. Cd in the soil was primarily in the ferromanganese oxidation state (S3) at 51.23%. This fraction will be indirectly absorbed by plants with a decrease in pH and the release of TM ions under reducing conditions, resulting in a potential ecological risk.

#### 3.2.4. Vegetables

**Figure 6:**
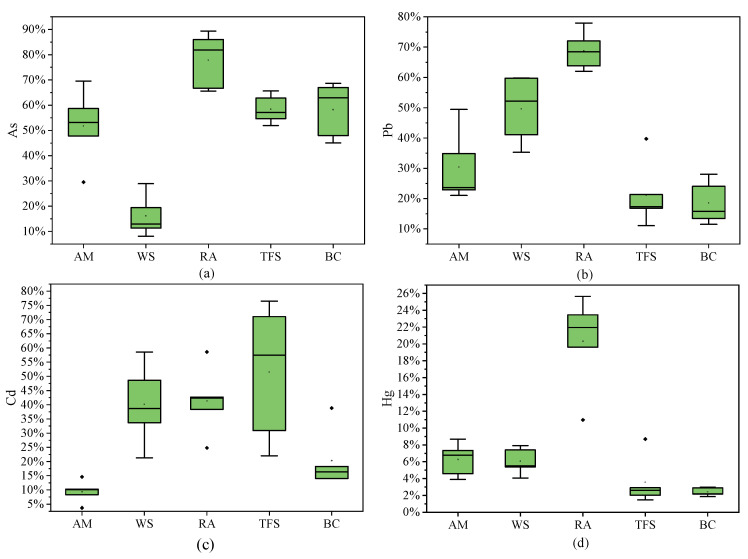
Bioaccessibility of 4 TMs in vegetables (BA_V_): As (**a**), Pb (**b**), Cd (**c**), Hg (**d**).

The bioaccessibility of different TMs during the stomach phase (BAV) in vegetables is depicted in Figure 6. For As, radish exhibited higher bioaccessibility compared to amaranth, cabbage, chard, and bok choy. In contrast, water spinach had significantly lower bioaccessibility of As, highlighting a notable difference in As uptake among these vegetables. For Pb, radish displayed the highest bioaccessibility, whereas bok choy had relatively lower bioaccessibility. For Cd, the highest bioaccessibility of Cd was found in radish, and the lowest was found in amaranth, indicating that the uptake of Cd in the stomach was significantly lower than that of other vegetables. For Hg, the highest and most significant bioaccessibility of Hg was found in radish, while the mean values of Hg were close to those of amaranth and water spinach, tender flower stalk, and bok choy, respectively, and the lowest was in bok choy. Overall, the bioaccessibility of TMs in radish was significantly higher than that of amaranth, water spinach, tender flower stalk, and bok choy for most metals, and in combination with the previous analyses, despite having lower total TM concentrations, radish exhibited greater bioaccessibility, suggesting that TMs in radish are more readily absorbed in the stomach.

The gastrointestinal bioaccessibility of As, Pb, Cd, and Hg in various vegetables is presented in Table 4. After simulated gastric digestion, the bioaccessibility of these TMs significantly decreased in the intestinal phase, indicating higher bioaccessibility and absorption potential in the gastric phase. For amaranth (AM), the bioaccessibility of As was the highest, indicating that As in AM is the most readily absorbed by the human body. For water spinach (WS), Pb was the most bioaccessible, indicating that Pb from WS is the most readily absorbed by humans. For radish (RA), As, Cd, and Pb showed higher bioaccessibility in the stomach compared to Hg, with As having the highest absorption rate, implying that radish As is particularly absorbable. For tender flower stalk (TFS), the absorption of As in them was the largest and Hg was the smallest, indicating that As in TFS was most easily absorbed by the human body, while much of Hg could not be absorbed. In the case of bok choy (BC), the amount of As absorbed in the stomach and intestine was the highest, which was more easily absorbed by the human body.

In summary, the degree of release of TMs from different types of vegetables in the human body was significantly different, which might be caused by the differences in vegetable varieties. Overall, the total bioaccessibility of the gastric and intestinal stages was ranked as As > Pb > Cd > Hg, in which the bioaccessibility of 3 TM elements, As, Cd and Pb, was elevated during the gastric phase and diminished during the intestinal phase, consistent with findings by Li Yi et al. This may be since the simulated fluid in the gastric stage has a lower pH value, and the enzyme activity is higher in the acidic environment, which makes it easier to release TM elements from it, thus making the bioaccessibility of TM elements in the gastric fluid high, whereas in the intestinal stage it is easier for adsorption and precipitation to occur, so that the released TMs can be immobilized and passivated, resulting in the release of the lower amount of TM elements in intestinal stage.

### 3.3. Bioaccessibility-Based Fuzzy Health Risk Assessment of Multimedia Environmental TM Exposure

#### 3.3.1. Ingestion Exposure

(1) Non-carcinogenic risks

The results of the fuzzy non-carcinogenic risk evaluation of the oral ingestion route are presented in Table 5. For soil, the non-carcinogenic risks of various metals through oral ingestion were ordered as follows: 1 > As [2.20 × 10^−3^, 1.10 × 10^−2^] > Cd [6.60 × 10^−4^, 3.30 × 10^−3^] > Hg [3.21 × 10^−4^, 1.81 × 10^−3^] > Pb [1.89 × 10^−4^, 9.43 × 10^−4^]. This indicates that As poses the highest non-carcinogenic risk. However, the non-carcinogenic risks of all soil TMs were still far lower than the acceptable risk threshold (HQ = 1). For dust, the total non-carcinogenic risks of various metals via oral ingestion were ranked as follows: Cd [2.85 × 10^−2^, 3.13] > Ni [7.51 × 10^−3^, 1.3] > As [8.54 × 10^−2^, 2.83 × 10^−1^] > Pb [8.09 × 10^−3^, 4.78 × 10^−2^]. Here, Cd presented the highest non-carcinogenic risk, next is Ni, with some exceeding acceptable risk threshold (HQ = 1), followed by As, Pb, all of which were below 1. For vegetables, the non-carcinogenic risks of the 4 TMs in all vegetables followed the same pattern: As > Cd > Pb > Hg. Among them, the health risks of amaranth and tender flower stalk were lower than those of the other 3 kinds, possibly due to the higher intake of light-colored vegetables.

In the single route of oral ingestion, the fuzzy non-carcinogenic risk associated with vegetables was substantially higher than that of soil and dust. For the total fuzzy non-carcinogenic risk of single elements, Cd [1.86 × 10^−1^, 3.33] > As [8.69 × 10^−1^, 1.25] > Pb [4.61 × 10^−2^, 5.63 × 10^−2^] > Hg [1.07 × 10^−2^, 1.45 × 10^−2^]. In addition, the non-carcinogenic risks of TMs in soil and vegetables were all below 1, except for Cd in dust. This indicates that the TMs ingested by local farmers through soil, dust, and vegetables pose no potential health risks to their bodies.

(2) Carcinogenic risks

The carcinogenic risk of TMs via oral ingestion is presented in Table 6. In terms of the cumulative carcinogenic risk levels of single-element soil, dust, and homegrown vegetables for orally ingested TM exposures, the total carcinogenic risk of the Pb, Cd, and As exposure pathways was As [1.92 × 10^−3^, 2.38 × 10^−3^] > Cd [2.98 × 10^−5^, 3.67 × 10^−5^] > Pb [7.92 × 10^−7^, 1.48 × 10^−6^], with As being the element with the highest cumulative carcinogenic risk among them.

Regarding terms of exposure media, the carcinogenic risk across the 3 media showed that vegetables > dust ≥ soil misuse, so edible vegetables should be prioritized as a control exposure media. When considering evaluating vegetable consumption, the carcinogenic risk followed the order CR (As) > CR (Cd) > CR (Pb) in almost all vegetables. From the perspective of total multi-elemental carcinogenic risk control, the total carcinogenic risk of TMs in the 5 vegetables in the following order: 1 > water spinach [8.44 × 10^−4^, 1.03 × 10^−3^] > bok choy [4.58 × 10^−4^, 5.60 × 10^−4^] > radish [2.50 × 10^−4^, 3.05 × 10^−4^] > amaranth [2.15 × 10^−4^, 2.63 × 10^−4^] > tender flower stalk [1.73 × 10^−4^, 2.11 × 10^−4^] > 1.00 × 10^−4^. The total carcinogenicity risk of all types of vegetables exceeded 1.00 × 10^−4^, which may be a risk of TM carcinogenicity, and the highest total carcinogenicity risk was found in water spinach, which needs to be taken seriously.

#### 3.3.2. Inhalation Exposure

(1) Non-carcinogenic risks

The results of the fuzzy non-carcinogenic risk evaluation based on morphology and lung fluid simulation for TMs As, Cd, Pb, Hg, and Ni exposed via the respiratory route are shown in Table 7. Among the elements assessed, the non-carcinogenic risks were ordered as follows: As [1.05 × 10^−1^, 1.17 × 10^+2^] > Cd [1.39 × 10^−2^, 9.84] > Ni [8.61 × 10^−3^, 5.34] > Pb [3.43 × 10^−3^, 4.05 × 10^−3^] > Hg [5.00 × 10^−5^, 1.26 × 10^−4^], notably, the non-carcinogenic risks of As, Cd, and Ni in certain scenarios exceeded the acceptability threshold (HQ = 1), suggesting that there is some non-carcinogenic risk in this scenario. Across the 3 media of soil, dust, and outdoor particulate matter (PM), the non-carcinogenic risks followed the order: airborne PM > dust > soil, and therefore, priority should be given to controlling the inhalation of particulate matter in outdoor air.

For soil media, As posed the highest non-carcinogenic risk, which was still well below the risk acceptability threshold (HQ = 1). For dust media, As had the highest non-carcinogenic risk, followed by Cd, Pb, and Ni, which were all below the risk acceptability threshold (HQ = 1).

The non-carcinogenic risk of TMs in PM2.5 to adults in descending order is: As [6.47 × 10^−2^, 7.94 × 10^+1^] > Cd [2.96 × 10^−3^, 4.24] > Ni [1.83 × 10^−3^, 2.58] > Pb [1.38 × 10^−3^, 1.51 × 10^−3^]. For PM10, the non-carcinogenic risk sequence was As [3.74 × 10^−2^, 3.78 × 10^+1^] > Cd [9.74 × 10^−3^, 5.60] > Ni [6.78 × 10^−3^, 2.76] > Pb [1.93 × 10^−3^, 2.24 × 10^−3^]. In terms of PM2.5 compared with PM10, As, Cd, Ni, and Pb in PM10 posed higher non-carcinogenic risks than those in PM2.5. In conclusion, a total of 3 metal elements, namely Cd, As, and Ni, in both PM2.5 and PM10, were identified as TMs with certain non-carcinogenic risks.

(2) Carcinogenic risks

The carcinogenic risk of TMs obscured by inhalation is shown in Table 8. For 3 exposure media, soil, dust, and PM, the carcinogenic risk posed by TMs, As [1.40 × 10^−8^, 4.35 × 10^−8^] ≈ Cd [2.08 × 10^−8^, 5.21 × 10^−8^] < 1.00 × 10^−6^ ≈ Ni [1.69 × 10^6^, 1.90 × 10^−6^], this finding indicates that respiratory exposure to these TMs in each medium does not pose a significant carcinogenic risk.

#### 3.3.3. Dermal Contact Exposure

(1) Non-carcinogenic risks

The non-carcinogenic risk values for dust and soil TMs in QCD by dermal exposure route were calculated via the health risk assessment model and are presented in Table 9. For adults, the non-carcinogenic risks of 3 TMs across all dust sampling sites in QCD ranged from 6.40 × 10^−5^ to 2.23 × 10^−3^, which did not exceed the corresponding thresholds for adults, and the overall non-carcinogenic levels of the various TMs in dust in QCD were low. The non-carcinogenic risk of dermal exposure to farmland soil in QCD was significantly below the acceptable risk threshold, and the non-carcinogenic risk contribution of various TMs to the human body was Cd > As > Hg > Pb > Ni.

(2) Carcinogenic risks

The health carcinogenic risk values for As in dust and soil from the QCD, evaluated via the dermal exposure route, indicated that the risk ranged from 9.87 × 10^−9^ to 1.12 × 10^−8^ for dust and from 7.46 × 10^−6^ to 8.05 × 10^−6^ for soil. The total carcinogenic risk through dermal contact was between 7.47 × 10^−6^ and 8.06 × 10^−6^. These results demonstrate that the carcinogenic risk posed by arsenic via the dermal pathway is low, and As in the QCD does not present a significant health risk to humans through this single route of exposure.

#### 3.3.4. Comprehensive Review of Multi-Pathway TM Health Risk Assessment

After analyzing the health risks tied to TMs in the 4 media of soil, dust, atmospheric particulate matter and locally grown vegetables in the QCD based on the above data, the evaluation coefficients of the non-carcinogenic risks of TMs through the 3 exposure routes of ingestion, inhalation and dermal contact can be derived to characterize the contribution of different exposure routes to overall non-carcinogenic risk, as detailed in Table 10.

The findings indicated that for adult residents of the chemical zone along the river in QCD, the highest exposure risk to Pb and Hg was through ingestion, followed by inhalation and dermal contact. This was because vegetables and a minimal quantity of soil dust were ingested, resulting in the highest risk of lead entering the human body through this route. Therefore, food safety must prioritize the issue of Pb content exceeding the standard. The highest risk of exposure by inhalation was observed for Cd, Ni, and 3 other elements, followed by ingestion and dermal contact. The primary health concern for inhalation was TMs associated with particulate matter. To mitigate the risk of exposure to TMs in the inhalation pathway of the human body, air particulate matter samplers have been set up in the downtown area of the chemical zone along the river in QCD. Sampling is conducted in different seasons to obtain representative data. The treatment of air particulate matter still needs to be persistently carried out to reduce inhalation-related TM exposure of the human body. Furthermore, the carcinogenic risk from the ingestion route is notably higher than that from inhalation and dermal contact. This contrasts with the non-carcinogenic risk assessment, where the risk of both the ingestion and inhalation routes exceeded the threshold. This is because the primary exposure medium in the inhalation pathway is atmospheric particulate matter, and different TMs in atmospheric particulate matter of varying particle sizes exhibit disparate bioaccessibility. As, Cd, and Ni in PM2.5, it was acknowledged that PM10 was more bioavailable than those in PM5 and TSP. Furthermore, the combined differences in the entry into the human body of particulate matter of different particle sizes result in variations in the non-carcinogenic risk under the inhalation pathway.

#### 3.3.5. Comprehensive Risk Management Policy

Beyond the pathway-specific findings, this study enables synthesized risk prioritization essential for effective environmental management. When integrating the magnitude of health risks with the scale of population exposure, the primary threats can be ranked as follows: The highest priority should be assigned to controlling As and Cd in leafy vegetables, particularly water spinach and bok choy, due to their significantly elevated carcinogenic risks and widespread consumption among local residents. The following 3 measures are recommended to be implemented: (1) relocating vegetable cultivation away from downwind areas of industrial complexes and implementing vegetation buffer zones using high-efficiency particulate-capturing species around industrial parks, complemented by vertical green barriers; (2) promoting low-accumulator crops such as radish to replace high-accumulator species and establishing mandatory pre-harvest screening and certification protocols for As, Cd, and Pb in agricultural products; and (3) addressing Cd and Ni in PM10, given their notable non-carcinogenic risks via inhalation affecting the general population in the industrial zone and enforcing real-time dust monitoring and high-efficiency baghouse dust collectors in critical industrial processes.

## 4. Conclusions

This study systematically assessed the health risks of TM exposure in the QCD by integrating fuzzy mathematical theory with multimedia bioaccessibility measurements. In contrast to traditional methods that rely solely on total metal concentrations, the novel application of a bioaccessibility-based fuzzy risk assessment model uniquely identified the ingestion of locally grown vegetables as the predominant exposure pathway. Specifically, the cumulative carcinogenic risk for residents consuming Locally grown vegetables exceeded the acceptable threshold (1.00 × 10^−6^), with As ([1.92 × 10^−3^, 2.37 × 10^−3^]) presenting the highest risk, followed by Cd and Pb. Among the vegetables analyzed, water spinach and bok choy were identified as the primary contributors to the cumulative carcinogenic risk. In comparison, inhalation exposure via soil, dust, and atmospheric particulates generally remained below carcinogenic risk thresholds, although non-carcinogenic risks associated with As, Cd, and Ni in fine particulate matter (PM2.5 and PM10) warrant continued attention. Dermal contact risks were consistently lower than those of ingestion. Based on these findings, the following targeted risk management strategies are recommended: Based on these findings, the targeted measures including relocating vegetable planting areas, promoting cultivation of low-enrichment crops, building vegetation buffer zones around the industrial park, etc., were proposed. For future methodological advances, machine learning algorithms, such as random forest neural networks, etc., could be employed to predict heavy metal bioaccessibility based on soil properties and total metal concentrations, thereby minimizing reliance on labor-intensive laboratory analyses and enabling much efficient, dynamic risk monitoring.

## Figures and Tables

**Table 1:** Latitude and longitude of atmospheric particulate matter sampling sites.

Sites	Latitude	Longitude
1	30°39′24.51″	114°25′40.06″
2	30°36′43.17″	114°25′55.58″
3	30°34′47.98″	114°27′03.46″
4	30°36′32.00″	114°28′43.16″

**Table 2:** Descriptive statistics of TM content in the dust (mg·kg^−1^).

	As	Cd	Pb	Ni
Mean	3.88	0.53	78.41	33.66
Min	2.47	0.31	18.63	13.50
Max	7.62	1.30	250.36	151.81
Standard deviation	1.65	0.31	79.45	37.67
Coefficient of variation	42%	58%	101%	107%
Hubei Province’s background values ^a^	12.3	0.17	26.7	29
Risk screening values ^b^	30, 25	0.3, 0.6	120, 170	100, 190

^a^ The arithmetic mean of the background values of Hubei Province in China’s Soil Elemental Background Values (China Environmental Monitoring General Station, 1990). ^b^ According to the standard [51], risk screening values were selected based on pH values, with the first number representing drylands with 6.5 < pH ≤ 7.5 and the second value representing soils with pH > 7.5.

**Table 3:** Descriptive statistics of TM content in the agricultural soils (mg·kg^−1^).

	As	Cd	Pb	Hg
Mean	3.84	0.24	18.82	0.29
Min	2.15	0.10	7.60	0.13
Max	5.11	0.65	67.30	0.57
Standard deviation	0.86	0.13	13.58	0.11
Coefficient of variation	23%	55%	72%	38%
Hubei Province background values ^a^	12.3	0.17	26.7	0.08
Risk screening values ^b^	30, 25	0.3, 0.6	120, 170	2.4, 3.4

^a,b^ Same meaning as Table 2.

**Table 4:** Gastrointestinal bioaccessibility of 4 TMs in various types of vegetables (mean values).

Vegetable Varieties	As		Cd		Pb		Hg	
	Gastric Stage	Enteric Phase	Gastric Stage	Enteric Phase	Gastric Stage	Enteric Phase	Gastric Stage	Enteric Phase
Amaranth	51.74%	17.67%	9.38%	2.36%	30.39%	1.73%	6.26%	2.99%
Water spinach	16.13%	7.90%	40.17%	7.90%	49.61%	6.18%	6.05%	2.82%
Radish	77.90%	21.03%	41.36%	17.02%	68.86%	31.83%	20.32%	21.95%
Tender flower stalk	58.45%	8.60%	51.59%	6.04%	21.24%	2.70%	3.54%	2.41%
Bok choy	58.30%	13.09%	20.32%	3.56%	18.55%	1.61%	2.42%	1.57%

**Table 5:** Assessment of the fuzzy non-carcinogenic risk of TMs via oral ingestion.

Exposure Medium		As	Cd	Pb	Hg	Ni
Soil		[2.20 × 10^−3^, 1.10 × 10^−2^]	[6.60 × 10^−4^, 3.30 × 10^−3^]	[1.89 × 10^−4^, 9.43 × 10^−4^]	[3.21 × 10^−4^, 1.81 × 10^−3^]	NA
Dust		[8.54 × 10^−2^, 2.83 × 10^−1^]	[2.85 × 10^−2^, 3.13]	[8.09 × 10^−3^, 4.78 × 10^−2^]	NA	[7.51 × 10^−3^, 1.3]
Vegetables	Amaranth	[8.67 × 10^−2^, 1.06 × 10^−1^]	[1.72 × 10^−2^, 2.10 × 10^−2^]	[5.66 × 10^−3^, 6.91 × 10^−3^]	[8.08 × 10^−4^, 9.88 × 10^−4^]	NA
	Water spinach	[3.40 × 10^−1^, 4.15 × 10^−1^]	[6.85 × 10^−2^, 8.37 × 10^−2^]	[2.08 × 10^−2^, 2.54 × 10^−2^]	[3.56 × 10^−3^, 4.35 × 10^−3^]	NA
	Radish	[1.01 × 10^−1^, 1.23 × 10^−1^]	[1.64 × 10^−2^, 2.01 × 10^−2^]	[5.32 × 10^−3^, 6.50 × 10^−3^]	[3.60 × 10^−3^, 4.40 × 10^−3^]	NA
	Tender flower stalk	[6.90 × 10^−2^, 8.44 × 10^−2^]	[2.22 × 10^−2^, 2.71 × 10^−2^]	[3.63 × 10^−3^, 4.44 × 10^−3^]	[7.90 × 10^−4^, 9.65 × 10^−4^]	NA
	Bok choy	[1.85 × 10^−1^, 2.26 × 10^−1^]	[3.27 × 10^−2^, 3.99 × 10^−2^]	[1.06 × 10^−2^, 1.30 × 10^−2^]	[1.59 × 10^−3^, 1.94 × 10^−3^]	NA
Total		[8.69 × 10^−1^, 1.25]	[1.86 × 10^−1^, 3.33]	[4.61 × 10^−2^, 5.63 × 10^−2^]	[1.07 × 10^−2^, 1.45 × 10^−2^]	[7.51 × 10^−3^, 1.3]

NA: not available due to the lack of evidence of toxicity.

**Table 6:** Assessment of the fuzzy carcinogenic risk of TMs via oral ingestion.

Exposure Medium		As	Cd	Pb
Soil		[2.39 × 10^−6^, 1.72 × 10^−5^]	[1.06 × 10^−8^, 6.06 × 10^−8^]	[1.32 × 10^−8^, 8.13 × 10^−8^]
Dust		[3.67 × 10^−6^, 2.53 × 10^−5^]	[3.57 × 10^−8^, 2.01 × 10^−8^]	[9.46 × 10^−8^, 5.59 × 10^−7^]
Vegetables	Amaranth	[2.12 × 10^−4^, 2.59 × 10^−4^]	[3.26 × 10^−6^, 3.98 × 10^−6^]	[8.41×10^−8^, 1.03×10^−7^]
	Water spinach	[8.31 × 10^−4^, 1.02 × 10^−3^]	[1.30 × 10^−5^, 1.59 × 10^−5^]	[3.09×10^−7^, 3.78×10^−7^]
	Radish	[2.46 × 10^−4^, 3.01 × 10^−4^]	[3.12 × 10^−6^, 3.82 × 10^−6^]	[7.91×10^−8^, 9.67×10^−8^]
	Tender flower stalk	[1.69 × 10^−4^, 2.06 × 10^−4^]	[4.21 × 10^−6^, 5.14 × 10^−6^]	[5.41×10^−8^, 6.61×10^−8^]
	Bok choy	[4.52 × 10^−4^, 5.53 × 10^−4^]	[6.21 × 10^−6^, 7.59 × 10^−6^]	[1.58×10^−7^, 1.94×10^−7^]
Total		[1.92 × 10^−3^, 2.38 × 10^−3^]	[2.98 × 10^−5^, 3.67 × 10^−5^]	[7.92 × 10^−7^, 1.48 × 10^−6^]

**Table 7:** Assessment of the fuzzy non-carcinogenic risk of TMs via inhalation.

Exposure Medium	As	Cd	Pb	Hg	Ni
Soil	[1.18 × 10^−3^, 3.77 × 10^−3^]	[3.37 × 10^−4^, 8.55 × 10^−4^]	[1.41 × 10^−5^, 3.84 × 10^−5^]	[5.00 × 10^−5^, 1.26 × 10^−4^]	NA
Dust	[1.81 × 10^−3^, 5.55 × 10^−3^]	[1.13 × 10^−3^, 2.83 × 10^−3^]	[1.01 × 10^−4^, 2.64 × 10^−4^]	NA	[5.69 × 10^−5^, 8.17 × 10^−5^]
PM2.5	[6.47 × 10^−2^, 7.94 × 10^+1^]	[2.96 × 10^−3^, 4.24]	[1.38 × 10^−3^, 1.51 × 10^−3^]	NA	[1.83 × 10^−3^, 2.58]
PM10	[3.74 × 10^−2^, 3.78 × 10^+1^]	[9.47 × 10^−3^, 5.60]	[1.93 × 10^−3^, 2.24 × 10^−3^]	NA	[6.78 × 10^−3^, 2.76]
Total	[1.05 × 10^−1^, 1.17 × 10^+2^]	[1.39 × 10^−2^, 9.84]	[3.43 × 10^−3^, 4.05× 10^−3^]	[5.00 × 10^−5^, 1.26 × 10^−4^]	[8.67 × 10^−3^, 5.34]

**Table 8:** Assessment of the fuzzy carcinogenic risk of TMs obscured by inhalation.

Exposure Medium	As	Cd	Ni
Soil	[5.51 × 10^−9^, 1.76 × 10^−8^]	[4.77 × 10^−9^, 1.21 × 10^−8^]	NA
Dust	[8.45 × 10^−9^, 2.59 × 10^−8^]	[1.60 × 10^−8^, 4.00 × 10^−8^]	[4.57 × 10^−10^, 6.56 × 10^−10^]
PM_2.5_	[4.35 × 10^−8^, 8.89 × 10^−8^]	[4.06 × 10^−8^, 1.34 × 10^−6^]	[5.17 × 10^−7^, 5.74 × 10^−7^]
PM_10_	[6.81 × 10^−8^, 9.71 × 10^−6^]	[6.77 × 10^−8^, 6.54 × 10^−6^]	[1.17 × 10^−6^, 1.33 × 10^−6^]
Total	[1.40 × 10^−8^, 4.35 × 10^−8^]	[2.08 × 10^−8^, 5.21 × 10^−8^]	[1.69 × 10^−6^, 1.90 × 10^−6^]

**Table 9:** Assessment of the fuzzy non-carcinogenic risk of TMs via the dermal route of exposure.

Exposure Medium	As	Cd	Pb	Hg	Ni
Dust	[6.40 × 10^−5^, 7.27 × 10^−5^]	[2.58 × 10^−4^, 9.12 × 10^−4^]	[5.48 × 10^−4^, 2.23 × 10^−3^]	NA	[2.03 × 10^−4^, 2.92 × 10^−4^]
Soil	[4.96 × 10^−3^, 2.69 × 10^−2^]	[1.50 × 10^−2^, 2.69 × 10^−2^]	[2.91 × 10^−3^, 5.35 × 10^−4^]	[1.63 × 10^−2^, 1.89 × 10^−2^]	NA
Total	[5.02 × 10^−3^, 2.70 × 10^−2^]	[1.53 × 10^−2^, 2.78 × 10^−2^]	[3.45 × 10^−4^, 2.77 × 10^−3^]	[1.63 × 10^−2^, 1.89 × 10^−2^]	[2.03 × 10^−4^, 2.92 × 10^−4^]

**Table 10:** Contribution of 3 exposure routes to the non-carcinogenic risk of TMs.

Exposure Route	As	Cd	Pb	Ni	Hg
Ingestion	16.87%	29.33%	99.95%	38.79%	99.95%
Inhalation	81.89%	70.30%	0.00%	61.21%	0.05%
Dermal contact	1.24%	0.37%	0.05%	NA	0.00%

## Data Availability

The datasets used and/or analyzed during the current study are available from the corresponding author on reasonable request.

## Supplementary Materials

The following supporting information can be downloaded at: https://www.mdpi.com/article/10.3390/toxics13100861/s1, Table S1: Sampling equipment and portable measuring instruments; Table S2: Table of toxicity parameters; Table S3: Calculated health risk parameters for multiple pathway exposures in the Qingshan riverine chemical zone; Standard references and Web references; Table S4: Carcinogenic risk assessment criteria; Table S5: Toxic metals in atmospheric particulate matter (C_PMM_); Table S6: Concentrations of toxic metals in atmospheric particulate matter (C_M_); Table S7: Toxic metal content of locally grown vegetables in North Lake area (mg·kg^−1^); Table S8: Levels of biologically accessible toxic metals in atmospheric particulate matter (C_PMBA_); Table S9: Concentrations of bioaccessible toxic metal in atmospheric particulate matter (C_BA_); Table S10: Bioaccessibility (BA_PM_) of toxic metal in atmospheric particulate matter; Table S11: Bioaccessibility of toxic metals in dust (BA_D_).
