# Supplementary material for: Bioaccessibility-Based Fuzzy Health Risk Assessment and Integrated Management of Toxic Metals Through Multimedia Environmental Exposure near Urban Industrial Complexes
Source: Toxics. 2025 Oct 11;13(10):861. doi: 10.3390/toxics13100861 (PMC12568006; doi:10.3390/toxics13100861)
Supplement: Supplementary file 1 [file toxics-13-00861-s001.zip › toxics-3903236-supplementary.pdf]

Supplementary Materials

Table S1 Sampling equipment and portable measuring instruments

Sample type	Name of instrument	Model	Number	Remark
PM sampler	Atmospheric particulate integrated sampler (Medium flow intelligent sampler)	FY-DQ101	2	PM2.5/PM10 /TSP Filter diameter Φ90mm±1mm
	Intelligent medium flow air total suspended particulate (TSP) sampler	TH-150F	2	TSP-PM10-PM5-PM2.5 Filter diameter Φ90mm±1mm
PM direct reading	PM2.5 measuring instrument	BLATN BR-HOL-1209	1	
Environmental conditions	GPS locator	Garmin eTrex301	1	

Supplementary of health risk assessment:

Health risk assessment calculations are based on "Exposure factors handbook of the U.S. Environmental Protection Agency" (USEPA, 1986), "Technical guidelines for risk assessment of soil contamination of land for construction" (HJ 25.3-2019), and "Exposure factors handbook of Chinese population: Adult".

Hazards identification and characterization:

Dose-effect assessment refers to the relationship between the content of toxic metal elements exposed to organisms and the biological response on the organisms. After quantitatively assessing the data of toxic metal pollutants, a quantitative estimation process is established to relate the probability of adverse health effects in exposed populations. The toxicity of non-carcinogens and carcinogens affects the dose-effect relationship. When the reference value of non-carcinogens is below the lowest threshold for health hazards, they will not pose a health hazard to humans, i.e., the non-carcinogenic reference dose (RfD). For carcinogens, the carcinogenic potency coefficient (SF) is used to represent the level of carcinogenic probability.

Table S2 Table of toxicity parameters [4]

	Method	Cd	As (inorganic)	Pb	Ni
RfD $mg \cdot kg^{-1} \cdot d^{-1}$	Oral ingestion	1.00E-03	3.00E-04	3.50E-03	2.00E-02
	Dermal contact	2.50E-05	3.00E-04	5.25E-04	/
	Inhalation	2.64E-06	3.96E-06	3.52E-03	2.11E-05
SF $kg \cdot d \cdot mg^{-1}$	Oral ingestion	3.80E-01	1.50	8.50E-03	/
	Dermal contact	/	1.50	/	/
	Inhalation	6.82	16.3	/	1.11E+00

Table S3 Calculated health risk parameters for multiple pathway exposures in the Qingshan riverine chemical zone

Medium	Parameters	Introduction	Unit	Value	
				Children	Adults
Dust	IngR	Rate of dust ingestion	mg·d^{-1}	200	100
	InhR	Rate of dust inhalation	m^{3}·kg^{-1}	7.6	20
	SA	Exposed skin area	cm^{2}	1600	4350
	AF	Amount of skin-adherent dust	mg·m^{-3}·d^{-1}	0.2	0.07
	EF	Exposure frequency for dust	day/year	350	350
	ED	Exposure duration for dust	year	6	24
	AT	Average time for carcinogenic effect	day	365*70	365*70
	ABS	Absorption factor	—	0.001	0.001
Agricultural soil	OSIR	Daily oral ingestion rate of soils	mg·d^{-1}		$\widetilde{\boldsymbol{OSIR}}^{\boldsymbol{\alpha}}$[20,100]
	PM	Atmospheric concentrations of PM	mg·m^{-3}		0.095
	DAIR	Daily air inhalation rate	m^{3}·d^{-1}		$\widetilde{\mathbf{DAIR}}$(12.3,16,19.6)
	SER	Skin exposure ratio	%		$\widetilde{\mathbf{SER}}$(18,32)
	SSAR	Adherence rate of soil on skin	mg·cm^{-2}		0.2
	ED	Exposure duration for soil	year		16-74.87
	EF	Exposure frequency for soil	day		365
	ABS_o	Absorption factor of oral ingestion	—		1
	PIAF	Retention fraction of inhaled particulates in body	—		0.75

Continued **Table S3** Calculated health risk parameters for multiple pathway exposures in the Qingshan riverine chemical zone.

Medium	Parameters	Introduction	Unit	Value	
				Children	Adults
Agricultural soil	f_{spo}	Fraction of soil-borne particulates in outdoor air	—		0.5
	EFO	Outdoor exposure frequency	%		$\widetilde{\mathbf{EFO}}$(4.2,14.9,34.7)
	f_{spi}	Fraction of soil-borne particulates in indoor air	—		0.8
	EFI	Indoor exposure frequency	%		1-EFO
Locally grown vegetables	IR[a]	Intake rate for light-color vegetables	$g{\cdot}d^{-1}$		287.9, $\widetilde{\boldsymbol{IR}}$[259.1,316.7]
		Intake rate for other vegetables	$g{\cdot}d^{-1}$		Need to discuss
	ED	Exposure duration for vegetables	year		74.87
	EF[b]	Exposure frequency for vegetables	year		Need to discuss
	RSP	Rate of self-production for light-color vegetables	%		95.35%
Composite indicators	BW	Average body weight	kg	15.9	56.9
	AT	Average time for non-carcinogenic effect	day	365*ED	365*ED

[a] Intake rate for light-coloured vegetables (IR): The average daily consumption of vegetables classified as dark-coloured and light-coloured vegetables according to the Manual is provided separately, and after taking the value of 10% interval, the IR of choy and amaranth is [75.2, 91.9], and that of bok choy, radish and water spinach is [259.1, 316.7] $g{\cdot}day^{-1}$.

[b] Exposure frequency for vegetables (EF): since the five vegetables sampled were all seasonal, the EF was set to the maximum possible value, "365 × 0.5 days".

Table S4 Carcinogenic risk assessment criteria

Risk level		Risk value
Level I	Very low risk	<1.00E-06
Level II	Low risk	[1.00E-06,1.00E-05)
Level III	Low-medium risk	[1.00E-05,5.00E-05)
Level IV	Medium risk	[5.00E-05,1.00E-04)
Level V	Medium-high risk	[1.00E-04,5.00E-04)
Level VI	High risk	[5.00E-04,1.00E-03)
Level VII	Very high risk	>1.00E-03

Table S5 Toxic metals in atmospheric particulate matter (C_{PMM})

Metal		PM2.5 mg·kg^{-1}	PM5 mg·kg^{-1}	PM10 mg·kg^{-1}	TSP mg·kg^{-1}
Cd	Mean	4.18E+01	4.93E+01	4.27E+01	4.53E+01
	Min	5.40E+00	5.89E+00	4.61E+00	5.19E+00
	Max	2.54E+02	2.32E+02	2.00E+02	1.70E+02
	Winter Mean	5.49E+01	9.07E+01	5.90E+01	7.61E+01
	Spring Mean	1.22E+01	1.20E+01	1.15E+01	9.23E+00
	Summer Mean	4.97E+01	5.09E+01	5.17E+01	5.08E+01
	Autumn Mean	6.03E+01	3.97E+01	5.92E+01	3.23E+01
Ni	Mean	1.51E+02	1.22E+02	1.84E+02	1.30E+02
	Min	3.59E+01	4.23E+01	4.55E+01	4.99E+01
	Max	7.23E+02	3.47E+02	6.61E+02	4.44E+02
	Winter Mean	6.07E+01	6.76E+01	7.75E+01	7.00E+01
	Spring Mean	7.36E+01	6.79E+01	7.68E+01	5.25E+01
	Summer Mean	2.20E+02	2.44E+02	2.71E+02	2.84E+02
	Autumn Mean	2.75E+02	2.18E+02	3.44E+02	2.11E+02
As	Mean	1.95E+02	1.08E+02	1.47E+02	8.02E+01
	Min	3.47E+01	3.56E+01	2.96E+01	2.77E+01
	Max	1.05E+03	2.50E+02	4.95E+02	2.22E+02
	Winter Mean	6.61E+01	6.26E+01	6.26E+01	5.22E+01
	Spring Mean	8.89E+01	7.08E+01	6.76E+01	3.57E+01
	Summer Mean	2.53E+02	1.74E+02	1.93E+02	1.40E+02
	Autumn Mean	4.06E+02	2.06E+02	2.90E+02	1.43E+02
Pb	Mean	1.91E+03	1.20E+03	1.73E+03	1.10E+03
	Min	2.64E+02	2.84E+02	2.47E+02	2.43E+02
	Max	1.08E+04	3.66E+03	6.23E+03	3.93E+03
	Winter Mean	6.05E+02	6.57E+02	6.37E+02	6.06E+02
	Spring Mean	5.68E+02	4.93E+02	4.90E+02	2.94E+02
	Summer Mean	2.98E+03	2.64E+03	2.81E+03	2.52E+03
	Autumn Mean	3.92E+03	2.25E+03	3.41E+03	1.86E+03

Table S6 Concentrations of toxic metals in atmospheric particulate matter (C_M)

Metal		PM2.5 mg·m^{-3}	PM5 mg·m^{-3}	PM10 mg·m^{-3}	TSP mg·m^{-3}
Cd	Mean	6.71E-06	1.57E-05	1.00E-05	1.96E-05
	Min	1.45E-06	2.31E-06	2.01E-06	3.87E-06
	Max	9.83E-05	1.01E-04	1.02E-04	1.04E-04
	Winter Mean	1.99E-05	3.90E-05	2.83E-05	4.33E-05
	Spring Mean	2.52E-06	4.27E-06	3.85E-06	6.58E-06
	Summer Mean	3.18E-06	3.51E-06	4.86E-06	5.20E-06
	Autumn Mean	2.65E-06	4.31E-06	5.14E-06	6.41E-06
Ni	Mean	1.45E-05	2.29E-05	2.71E-05	3.68E-05
	Min	6.68E-06	1.27E-05	1.64E-05	2.27E-05
	Max	2.12E-05	3.18E-05	5.09E-05	5.52E-05
	Winter Mean	1.55E-05	2.44E-05	2.69E-05	3.57E-05
	Spring Mean	1.64E-05	2.54E-05	2.71E-05	4.18E-05
	Summer Mean	1.36E-05	1.50E-05	2.47E-05	2.68E-05
	Autumn Mean	1.17E-05	2.28E-05	2.95E-05	4.13E-05
As	Mean	1.73E-05	2.10E-05	2.15E-05	2.38E-05
	Min	8.07E-06	9.14E-06	1.03E-05	1.14E-05
	Max	2.90E-05	3.26E-05	3.41E-05	3.14E-05
	Winter Mean	1.70E-05	2.16E-05	2.09E-05	2.46E-05
	Spring Mean	1.80E-05	2.47E-05	2.15E-05	2.69E-05
	Summer Mean	1.60E-05	1.06E-05	1.83E-05	1.30E-05
	Autumn Mean	1.81E-05	2.28E-05	2.53E-05	2.85E-05
Pb	Min	8.51E-05	1.34E-04	1.03E-04	2.02E-04
	Max	2.23E-04	2.89E-04	4.27E-04	4.57E-04
	Winter Mean	1.58E-04	2.26E-04	2.20E-04	2.86E-04
	Spring Mean	1.19E-04	1.76E-04	1.61E-04	2.22E-04
	Summer Mean	1.88E-04	1.65E-04	2.61E-04	2.39E-04
	Autumn Mean	1.65E-04	2.41E-04	2.93E-04	3.67E-04
	Min	8.51E-05	1.34E-04	1.03E-04	2.02E-04

Table S7 Toxic metal content of local grown vegetables in North Lake area (mg·kg^{-1})

Vegetables		As	Cd	Pb	Hg
Amaranth	Mean	0.030	0.145	0.048	0.002
	Min	0.021	0.062	0.019	0.001
	Max	0.048	0.357	0.083	0.003
Water spinach	Mean	0.140	0.033	0.029	0.003
	Min	0.038	0.022	0.01	0.002
	Max	0.190	0.046	0.041	0.003
Radish	Mean	0.007	0.006	0.004	0.001
	Min	0.006	0.005	0.002	0.000
	Max	0.007	0.008	0.009	0.002
Tender flower stalk	Mean	0.023	0.038	0.052	0.005
	Min	0.016	0.011	0.007	0.001
	Max	0.030	0.083	0.088	0.007
Bok choy	Mean	0.017	0.033	0.041	0.003
	Min	0.010	0.020	0.027	0.002
	Max	0.024	0.047	0.047	0.003

Table S8 Levels of biologically accessible toxic metals in atmospheric particulate matter (C_{PMBA})

Metal		PM2.5 mg·kg^{-1}	PM5 mg·kg^{-1}	PM10 mg·kg^{-1}	TSP mg·kg^{-1}
Cd	Mean	1.19E+01	9.06E+00	1.36E+01	1.25E+01
	Min	3.29E+00	4.12E+00	5.69E+00	4.63E+00
	Max	4.75E+01	1.20E+01	5.30E+01	1.66E+01
	Winter Mean	8.34E+00	1.17E+01	1.13E+01	1.64E+01
	Spring Mean	6.80E+00	6.00E+00	6.09E+00	4.63E+00
	Summer Mean	1.97E+01	—	2.37E+01	—
	Autumn Mean	8.55E+00	7.92E+00	9.83E+00	—
Ni	Mean	5.23E+01	7.34E+01	6.91E+01	6.01E+01
	Min	2.49E+00	1.56E+01	1.46E+01	1.78E+01
	Max	1.77E+02	1.04E+02	1.38E+02	1.02E+02
	Winter Mean	1.49E+01	2.12E+01	2.43E+01	2.64E+01
	Spring Mean	6.30E+00	—	1.57E+01	—
	Summer Mean	1.04E+02	9.67E+01	9.68E+01	8.62E+01
	Autumn Mean	5.78E+01	1.02E+02	8.40E+01	1.02E+02
As	Mean	1.24E+02	8.39E+01	1.08E+02	5.74E+01
	Min	1.70E+01	3.19E+01	2.93E+01	2.96E+01
	Max	4.59E+02	1.63E+02	2.48E+02	1.13E+02
	Winter Mean	3.34E+01	4.47E+01	4.12E+01	4.05E+01
	Spring Mean	2.32E+01	—	—	—
	Summer Mean	1.23E+02	5.59E+01	9.91E+01	3.54E+01
	Autumn Mean	2.52E+02	1.51E+02	1.65E+02	1.13E+02
Pb	Mean	5.26E+02	3.43E+02	4.67E+02	3.34E+02
	Min	1.35E+02	1.37E+02	1.28E+02	1.19E+02
	Max	2.04E+03	6.52E+02	1.24E+03	7.51E+02
	Winter Mean	2.34E+02	2.50E+02	2.57E+02	2.40E+02
	Spring Mean	1.49E+02	1.50E+02	1.40E+02	1.19E+02
	Summer Mean	5.73E+02	4.98E+02	5.76E+02	5.10E+02
	Autumn Mean	9.26E+02	5.21E+02	7.16E+02	4.07E+02

Table S9 Concentrations of bioaccessible toxic metal in atmospheric particulate matter (C_{BA})

Metal		PM2.5 mg·m^{-3}	PM5 mg·m^{-3}	PM10 mg·m^{-3}	TSP mg·m^{-3}
Cd	Mean	1.37E-06	2.70E-06	2.51E-06	6.81E-06
	Min	3.60E-07	5.51E-07	8.67E-07	4.44E-06
	Max	3.52E-06	6.17E-06	7.14E-06	1.15E-05
	Winter Mean	2.77E-06	4.46E-06	5.04E-06	8.00E-06
	Spring Mean	1.60E-06	2.90E-06	2.49E-06	4.44E-06
	Summer Mean	1.30E-06	NA	2.25E-06	NA
	Autumn Mean	6.23E-07	8.31E-07	1.08E-06	NA
Ni	Mean	4.61E-06	8.95E-06	1.02E-05	1.63E-05
	Min	3.88E-07	3.40E-06	1.89E-06	9.81E-06
	Max	1.11E-05	1.40E-05	1.82E-05	2.16E-05
	Winter Mean	4.04E-06	7.28E-06	9.01E-06	1.10E-05
	Spring Mean	1.78E-06	NA	5.63E-06	NA
	Summer Mean	7.00E-06	7.84E-06	1.26E-05	2.15E-05
	Autumn Mean	4.43E-06	1.17E-05	1.01E-05	2.16E-05
As	Mean	9.98E-06	1.23E-05	1.43E-05	1.69E-05
	Min	1.48E-06	2.15E-06	5.94E-06	8.86E-06
	Max	2.06E-05	2.18E-05	2.31E-05	2.41E-05
	Winter Mean	9.34E-06	1.52E-05	1.61E-05	1.74E-05
	Spring Mean	7.06E-06	NA	NA	NA
	Summer Mean	8.73E-06	4.30E-06	1.09E-05	8.86E-06
	Autumn Mean	1.36E-05	1.75E-05	1.76E-05	2.41E-05
Pb	Mean	4.62E-05	6.74E-05	6.68E-05	9.23E-05
	Min	1.58E-05	2.38E-05	2.05E-05	3.85E-05
	Max	8.04E-05	1.05E-04	1.19E-04	1.32E-04
	Winter Mean	6.28E-05	8.92E-05	9.25E-05	1.18E-04
	Spring Mean	4.46E-05	7.73E-05	6.56E-05	1.14E-04
	Summer Mean	3.75E-05	3.29E-05	5.54E-05	5.30E-05
	Autumn Mean	4.50E-05	5.94E-05	6.19E-05	8.22E-05

Table S10 Bioaccessibility (BA_{PM}) of toxic metal in atmospheric particulate matter

Metal		PM2.5	PM5	PM10	TSP
Cd	Mean	41.7%	29.2%	39.1%	36.7%
	Min	12.5%	14.8%	17.5%	27.8%
	Max	82.7%	61.5%	85.9%	52.5%
	Winter Mean	57.0%	23.5%	22.7%	28.8%
	Spring Mean	75.1%	61.5%	71.1%	52.5%
	Summer Mean	37.2%	—	44.3%	—
	Autumn Mean	21.8%	18.8%	23.4%	—
Ni	Mean	29.7%	43.3%	35.4%	49.5%
	Min	3.7%	26.8%	8.1%	34.3%
	Max	63.8%	93.8%	87.7%	78.6%
	Winter Mean	26.1%	32.5%	32.8%	35.0%
	Spring Mean	9.3%	—	21.6%	—
	Summer Mean	40.7%	26.8%	36.9%	—
	Autumn Mean	40.1%	62.4%	39.4%	78.6%
As	Mean	52.7%	53.3%	59.9%	65.7%
	Min	18.4%	23.6%	31.2%	52.0%
	Max	90.1%	81.0%	79.6%	84.7%
	Winter Mean	55.2%	50.9%	51.6%	52.0%
	Spring Mean	41.7%	81.0%	79.6%	84.7%
	Summer Mean	49.9%	38.6%	57.3%	60.5%
	Autumn Mean	65.2%	57.4%	59.7%	—
Pb	Mean	28.6%	31.5%	28.3%	31.9%
	Min	15.4%	17.2%	12.5%	15.4%
	Max	52.2%	53.1%	52.9%	52.5%
	Winter Mean	40.3%	40.2%	41.7%	40.7%
	Spring Mean	32.5%	35.6%	34.7%	40.1%
	Summer Mean	19.7%	19.6%	21.1%	21.7%
	Autumn Mean	27.1%	26.5%	22.2%	24.8%

Table S11 Bioaccessibility of toxic metals in dust (BA_D)

Sampling number	Point position	Cd	Ni	As	Pb
①	1	29.5%	15.8%	6.0%	2.3%
	2	50.6%	19.8%	6.5%	8.5%
	3	34.8%	13.6%	3.2%	2.1%
	4	33.0%	18.7%	2.6%	1.3%
②	1	37.4%	12.5%	5.1%	4.9%
	2	29.0%	5.2%	6.2%	9.0%
	3	39.5%	16.6%	3.8%	3.2%
	4	26.0%	18.5%	3.9%	3.0%
③	1	43.3%	12.5%	8.2%	2.2%
	2	58.3%	18.2%	10.6%	3.2%
	3	62.2%	24.9%	13.2%	3.9%
	4	55.8%	15.5%	8.7%	1.5%
④	1	44.1%	6.6%	6.6%	1.5%
	2	44.0%	7.3%	9.1%	1.3%
	3	36.9%	27.1%	2.7%	0.04%
	4	51.4%	12.1%	3.2%	1.8%
